# Subjective burden and perspectives of German healthcare workers during the COVID-19 pandemic

**DOI:** 10.1007/s00406-020-01183-2

**Published:** 2020-08-19

**Authors:** Victoria Kramer, Irina Papazova, Andreas Thoma, Miriam Kunz, Peter Falkai, Thomas Schneider-Axmann, Anke Hierundar, Elias Wagner, Alkomiet Hasan

**Affiliations:** 1 Department of Psychiatry, Psychotherapy and Psychosomatics, Medical Faculty, University of Augsburg, BKH Augsburg, Dr.-Mack-Str. 1, 86156 Augsburg, Germany; 2 Department of Psychiatry and Psychotherapy, LMU University Hospital, Munich, Germany; 3 Department of Medical Psychology and Sociology, Medical Faculty, University of Augsburg, Augsburg, Germany; 4 Department of Anaesthesiology, University of Rostock, Rostock, Germany

**Keywords:** Stress, COVID-19, Pandemic, Personal burden, Depression, Anxiety

## Abstract

Healthcare workers (HCW) face tremendous challenges during the COVID-19 pandemic. Little is known about the subjective burden, views, and COVID-19 infection status of HCWs. The aim of this work was to evaluate the subjective burden, the perception of the information policies, and the agreement on structural measures in a large cohort of German HCW during the COVID-19 pandemic. This country-wide anonymous online survey was carried out from April 15th until May 1st, 2020. 25 content-related questions regarding the subjective burden and other dimensions were evaluated. We evaluated different dimensions of subjective burden, stress, and perspectives using 5-point Likert-scale questions. Moreover, the individual COVID-19 infection status, the amount of people infected in circle of friends and acquaintances and the hours working overtime were assessed. A total of 3669 HCWs provided sufficient responses for analyses. 2.8% of HCWs reported to have been tested positive for COVID-19. Nurses reported in principle higher ratings on all questions of subjective burden and stress than doctors and other hospital staff. Doctors (3.6%) and nurses (3.1%) were more likely to be tested positive for COVID-19 than other hospital staff (0.6%, Chi_(2)_^2^ = 17.39, *p* < 0.0005). HCWs who worked in a COVID-19 environment reported higher levels of subjective burden and stress compared to all other participants. Working in a COVID-19 environment increased the likelihood to be tested positive for COVID-19 (4.8% vs. 2.3%, Chi_(1)_^2^ = 12.62, *p* < 0.0005) and the severity of the subjective burden. During the COVID-19 pandemic, nurses experience more stress than doctors. Overall, German HCWs showed high scores of agreement with the measures taken by the hospitals.

**Electronic supplementary material:**

The online version of this article (10.1007/s00406-020-01183-2) contains supplementary material, which is available to authorized users.

## Introduction

The COVID-19 outbreak in 2020 is the most devastating pandemic since the Spanish flu in 1918. Outbreaks of old and new infectious agents occur on a regular basis. The Zika virus epidemic, the 2009 flu pandemic (H1N1/09 virus), and the SARS epidemic between 2002 and 2004 were manifestations of viruses that received large attention by the media and politics. The SARS outbreak was previously described as a crisis unprecedented in terms of infectiousness and speed of spreading across the world [1]. In a larger context, the SARS epidemic was one example among many others, showing that healthcare workers (HCW) facing an infectious outbreak in the front line expose themselves to a substantial risk of developing mental health problems [2].

One recently published meta-analysis comprising 61 studies mainly conducted in hospital settings on the Asian continent investigated mental-health status of HCWs during an infectious disease outbreak. Bearing in mind that most studies did not use validated instruments to evaluate the mental-health status, this meta-analysis showed an increased pooled prevalence for anxiety, depression, acute stress, and post-traumatic stress disorders in HCWs [2]. In this context, a systematic review concluded that poor mental-health outcomes among HCWs were associated with exposure to high-risk environments, with strict regulations involving quarantine, poor organizational support, role-related stressors, and a lack of subjectively perceived safety [1].

The recent COVID-19 pandemic significantly exceeds the aforementioned SARS pandemic in many aspects. It seems to be evident that this pandemic will have a tremendous impact on exposed HCWs. Starting in late 2019 in Wuhan, COVID-19 was defined to be a pandemic by the World Health Organization (WHO) on March 11, 2020. By the end of March, nearly all countries worldwide were struggling with the spread of the virus (course of events summarized by [2]). In the middle of June 2020, the WHO reported nearly 7.5 million COVID-19 infections all around the world [3].

The aforementioned meta-analysis [2] identified three studies evaluating the impact of the COVID-19 pandemic on HCWs’ mental health. One cross-sectional study among 1257 Chinese HCWs in 34 national hospitals involved in the management of COVID-19 patients reported a significant psychological burden, especially among female nurses. Furthermore, self-report ratings indicated increased symptoms of depression, anxiety, insomnia, and distress [4]. Another cross-sectional study from China compared 234 frontline nurses with 294 non-frontline nurses and showed a higher rate of vicarious traumatization among non-frontline nurses [5]. Moreover, one cross-sectional study with a 1-month observation period among 180 medical professionals from Wuhan involved in the management of COVID-19 reported high levels of stress-related symptoms that were associated with deteriorated sleep quality [6]. An online-study investigated anxiety symptoms among 85 Iranian nurses showed high rates of anxiety regarding their own well-being and of their respective families [7]. Related findings were reported in an online survey conducted among 1257 Italian HCWs [8]. These findings are in line with the findings from previous epidemic and pandemic outbreaks as detailed above. However, large-scale evaluations of the impact of the COVID-19 pandemic on mental health of HCWs in high-income economies and the differentiation between professional groups are sparse.

Germany has one of the worldwide leading healthcare systems. The system is mainly publicly financed in general without any upfront payments and covers for most people living in Germany. It covers inpatient and outpatient care for every diagnosis group as well as a broad array of preventive services. Especially, the high number of inpatient beds (6/1.000; e.g., US has 2.4/1.000 beds) and ICU beds (33.9/100.000) per inhabitant differentiates the German system from those from other high-income countries [9]. For example, Spain and Italy, both countries who faced tremendous COVID-19 outbreaks have 9.7 and, respectively, 8.6 ICU beds per 100.000 inhabitants [9]. Given that Germany was not confronted with any epidemic or pandemic for a very long period, little is known of the impact of such medical emergency on the mental health of HCWs.

## Methods

### Participants and survey

From April 15th 2020 until May 1st 2020, we conducted an online survey using a licensed LimeSurvey version 2.06 [10]. The survey was reviewed by the data protection officer of the University Hospital Munich (LMU Munich) and the local ethical committee (20–309 KB; waiver). The management boards of 35 university hospitals in Germany, of 58 other secondary and tertiary and secondary care hospitals in Germany and of all psychiatric hospitals organized in the Federal Director Conference were asked to distribute the survey link to their staff via mail. Moreover, the German Association for Psychiatry, Psychotherapy and Psychosomatics (~ 10.000 members), the German Society of Surgery (~ 20.000 members), and the German Interdisciplinary Association for Intensive and Emergency Medicine (~ 2.300 members) sent the survey link to their members. Cochrane Germany shared the survey link via their twitter channel (Cochrane Deutschland@Cochrane_DE).

### Survey structure

The survey was first developed by VK, AT, and AH and revised by EW, TSA, and MK. The survey was provided in German language and translated for this publication (see supplement). 5-point Likert-scale questions ranged from 1 (strongly disagree) to 5 (strongly agree). For the variable working experience, the response < 3 years was not exported correctly, since the answer categories were misleading. To avoid bias, this variable was excluded from further analyses.

### Statistical analyses

IBM SPSS for Windows (version 25) was used for statistical analyses. As 25 questions (question 9 – 33) were analysed, significance level was Bonferroni-adjusted to α = 0.05/25 = 0.002. Results of *p* > 0.002, but *p* < 0.05 were indicated as trends. Descriptive statistics include frequencies, mean, standard deviation (SD), minimum, maximum, sum, and median. All these parameters were applied for continuous variables and frequencies were applied to present dichotomous variables. Sample sizes smaller than 3669 indicate missing responses for the respective variables. Three-group comparisons between medical doctors (MD), nurses, and other hospital staff involved in patient care were conducted with Kruskal–Wallis tests. In the case of significant effects in the Kruskal–Wallis test, post hoc subgroup comparisons were performed using Mann–Whitney U tests (MWU). For post hoc comparisons, the significance level was adjusted to α = 0.05/25/3 = 0.000667. Results of *p* > 0.000667, but *p* < 0.0133 were indicated as trends. Two-group comparisons between professionals working in areas with high risk to be in contact with COVID-19 patients [emergency room (ER), intensive care unit (ICU), and COVID-19 special units] and all others were performed with MWU tests. Demographic differences between groups were tested with Chi-square tests, with one-way-ANOVAs (Welch–ANOVA if Levene’s test showed *p* < 0.05), and with Bonferroni-corrected or Games–Howell post hoc tests where appropriate.

## Results

### Survey

A total of 5822 participants opened the link to the survey during the assessment period. For the here presented analyses, all participants who did not reach question 9 of the questionnaire (commencement of content-based questions) (*N* = 479) were excluded. From this population (*n* = 5343), all persons not working in direct patient care (administration: *n* = 608; science: *n* = 240; other reason for not working in patient care: *n* = 199; missing data: *n* = 627) were excluded leaving 3669 participants. Due to potential biased values in the “average overtime” variable, *N* = 1 (reported -80 h/week) and *N* = 12 (reported > 30 h/week), a total of *N* = 13 subjects were excluded for this variable (extreme values that exceeded the 75%-quartile or were below the 25% quartile by more than ten times of the IQR).

### Demographic information

61.0% of the participants were female and our sample covered the complete age range of HCWs. The distribution of occupational groups included 11.7% residents, 8.6% head physicians/chief of departments, 23.8% physicians/board-certified physicians, 35.9% nurses, 8.4% psychologists, 4.5% social workers, and 7.1% working in other areas of patient care. 40.1% worked in university hospitals and 59.9% in non-university hospitals. 19.2% worked in the emergency room, ICU, or on a special COVID-19 ward. See Table 1 and Supplementary Table 1 for demographic information.

**Table 1:** Demographic characteristics of the complete sample

Item	*N* (%)
Gender	3645 (99.3)
Female	2225 (61.0)
Male	1415 (38.8)
Third	5 (0.1)
Age (years)	3659 (99.7)
18–30	699 (19.1)
31–40	963 (26.3)
41–50	757 (20.7)
51–60	925 (25.3)
> 60	315 (8.6)
Area of work	3669 (100.0)
Head Physician	316 (8.6)
Resident	428 (11.7)
Specialist	873 (23.8)
Nurse	1317 (35.9)
Psychologist	310 (8.4)
Social worker	164 (4.5)
Other	261 (7.1)
Area of hospital	3669 (100.0)
Ambulatory	601 (16.4)
Emergency room (ER)	188 (5.1)
Unit floor/ward	2362 (64.4)
COVID-19 ward	129 (3.5)
Intensive care unit (ICU)	389 (19.6)
ICU, ER or COVID-19 ward	3669 (100.0)
Yes	706 (19.2)
No	2963 (80.8)
Type of hospital	3617 (98.6)
University hospital	1449 (40.1)
Other	2168 (59.9)

### COVID-19-related demographic information

2.8% of all participants were tested positive for COVID-19 at the time of responding to the survey and 26.5% belonged to the COVID-19 risk group due to pre-existing medical conditions, age, or other factors. Participants worked on average 2.01 h overtime since the beginning of the COVID-19 pandemic (minimum = − 26; maximum =+ 30; Mdn = 0.00; 95% CI for the mean 1.87–2.15; SD = 4.07; *N* = 3414) treated on average 3.11 patients with COVID-19 (minimum = 0; maximum = 350; SD = 11.06; Mdn 0.00; 95% CI for the mean = 2.74–3.47; *N* = 3493), and reported to have on average 1.20 friends or family members tested positive for COVID-19 (minimum = 0; maximum = 50; Mdn = 0; 95% CI for the mean = 1.11–1.29; SD = 2.75; *N* = 3541).

### Overall response patterns

With regard to subjective burden, high ratings were reached regarding the questions of subjective mental stress (question 10), of worrying about the personal future (question 26), of worrying regarding the well-being of the family (questions 27) and of the fear to catch the virus and to pass it on to family or friends (question 29). On the other hand, low scores of agreement were reported with regard to a lack of time in personal life (question 24) and a reduced sleep quality (question 30). Evaluating structural factors of the hospitals, participants rated the information policy, the measures taken by the hospitals to provide safety equipment (question 19), the communication strategy (questions 20), and the preparation of the hospital for the pandemic (questions 22) mostly positive. Importantly, low ratings emerged for the question of feeling left alone by the employer (question 13) and most participants strongly agreed that they are willing to continue working in the healthcare system after the pandemic (question 33). See Table 2 for the descriptive statistics of all answers of the complete sample.

**Table 2:** Descriptive data of the complete sample for the 5-item Likert-scale questions

Item	Total	Strongly disagree	Disagree	Neutral	Agree	Strongly agree	*Mdn*	*M*	*(SD)*
	*N* (%)	*n* (%)	*n* (%)	*n* (%)	*n* (%)	*n* (%)			
The COVID-19 pandemic has led to an increase in my daily workload (Q9)	3652 (99.5%)	702 (19.2%)	739 (20.2%)	698 (19.1%)	925 (25.3%)	588 (16.1%)	3.0	2.99	(1.37)
Due to the COVID-19 pandemic I feel mentally strained (Q10)	3634 (99.0%)	336 (9.2%)	603 (16.6%)	645 (17.7%)	1417 (39.0%)	633 (17.4%)	4.0	3.39	(1.21)
My superiors/my employer informed me sufficiently about COVID-19 (Q11)	3615 (98.5%)	170 (4.7%)	528 (14.6%)	662 (18.3%)	1187 (32.8%)	1068 (29.5%)	4.0	3.68	(1.18)
Since the outbreak of the COVID-19 pandemic, the satisfaction with my job has worsened (Q12)	3612 (98.4%)	499 (13.8%)	665 (18.4%)	737 (20.4%)	1033 (28.6%)	678 (18.8%)	3.0	3.20	(1.32)
I feel left alone by my employer (Q13)	3597 (98.0%)	1066 (29.6%)	1055 (29.3%)	688 (19.1%)	558 (15.5%)	230 (6.4%)	2.0	2.40	(1.24)
I feel left alone by the responsible political decision-makers (Q14)	3590 (97.8%)	655 (18.2%)	1081 (30.1%)	728 (20.3%)	653 (18.2%)	473 (13.2%)	3.0	2.78	(1.30)
The measures taken by the hospital administration have been appropriate (in terms of supply with information, protective equipment, organization of work processes) (Q19)	3563 (97.1%)	317 (8.9%)	860 (24.1%)	542 (15.2%)	1220 (34.2)	624 (17.5%)	4.0	3.27	(1.25)
In my opinion, the communication related to COVID-19 that came from the management of the hospital has been appropriate (Q20)	3514 (95.8%)	206 (5.9%)	574 (16.3%)	630 (17.9%)	1223 (34.8%)	881 (25.1%)	4.0	3.57	(1.19)
I have the impression that my efforts at work during the COVID-19 pandemic are being appreciated by the management of the hospital (Q21)	3519 (95.9%)	329 (9.3%)	705 (20.0%)	977 (27.8%)	951 (27.0%)	557 (15.8%)	3.0	3.20	(1.20)
My hospital was/is well prepared with regard to the COVID-19 pandemic (Q22)	3526 (96.1%)	244 (6.9%)	582 (16.5%)	714 (20.2%)	1253 (35.5%)	733 (20.8%)	4.0	3.47	(1.19)
Due to the COVID-19 pandemic, I have significantly less time for my personal life (Q24)	3526 (96.1%)	1316 (37.3%)	872 (24.7%)	626 (17.8%)	453 (12.8%)	249 (7.3%)	2.0	2.28	(1.28)
My daily life has become more stressful due to the COVID-19 pandemic (Q25)	3517 (95.9%)	716 (20.4%)	725 (20.6%)	556 (15.8%)	935 (26.6%)	585 (16.6%)	3.0	2.99	(1.40)
Due to the COVID-19 pandemic, I am worrying more often about the future (Q26)	3525 (96.1%)	368 (10.4%)	672 (19.1%)	584 (16.6%)	1225 (35.3%)	656 (18.6%)	4.0	3.33	(1.27)
Due to the COVID-19 pandemic I am worrying more often about the well-being of my family (Q27)	3519 (95.9%)	158 (4.5%)	406 (11.5%)	371 (10.5%)	1368 (38.9%)	1216 (34.6%)	4.0	3.87	(1.14)
I am afraid of catching the Coronavirus myself (Q28)	3502 (95.4%)	698 (19.9%)	1047 (29.9%)	811 (23.2%)	633 (18.1%)	313 (8.9%)	3.0	2.66	(1.23)
I fear that due to my daily exposure with it at work, I could pass on the coronavirus to my friends or relatives (Q29)	3501 (95.4%)	309 (8.8%)	584 (16.7%)	495 (14.1%)	1129 (32.2%)	984 (28.1%)	4.0	3.54	(1.29)
Since the COVID-19 pandemic, I have been sleeping less well (Q30)	3514 (95.8%)	1332 (37.9%)	771 (21.9%)	553 (15.7%)	561 (15.7%)	297 (8.5%)	2.0	2.35	(1.35)
In my setting, patients not infected with COVID-19 are adequately taken care of despite the COVID-19 pandemic (Q31)	3426 (93.4%)	332 (9.7%)	856 (25.0%)	552 (16.1%)	988 (28.8%)	698 (20.4%)	3.0	3.25	(1.30)
In my hospital setting, COVID-19 positive patients are adequately taken care of (Q32)	3061 (83.4%)	234 (7.6%)	372 (12.2%)	742 (24.2%)	976 (31.9%)	737 (24.1%)	4.0	3.53	(1.20)
I will continue to work in the healthcare area after the COVID-19 pandemic (Q33)	3459 (94.3%)	60 (1.7%)	92 (2.7%)	156 (4.5%)	457 (13.2%)	2694 (77.9%)	5.0	4.63	(0.82)

### Contrasting MD, nurses, and other hospital staff

ANOVA showed significant differences between groups in the average hours of overtime per week (Welch test: F_(2, 2224.7)_ = 92.88, *p* < 0.0005) with more overtime hours among nurses (2.60 ± 4.30) and MDs (2.09 ± 4.44) compared to other staff (0.80 ± 2.04) (*p* < 0.0005 each). The contrast between nurses and MDs did not reach the adjusted significance threshold (*p* = 0.0079). Group differences were detected in the number of patients treated who were positive for COVID-19 (Welch test: F_(2, 2318.6)_ = 72.20, *p* < 0.0005). MDs (4.28 ± 14.81) treated numerically more patients than nurses (2.99 ± 7.40) (*p* = 0.008, not reaching the adjusted significance threshold) and other staff (0.65 ± 3.57) (*p* < 0.0005). Nurses treated more patients than other staff (*p* < 0.0005). Regarding the number of family members and friends having been tested positive, again, significant differences between groups differences were detected (Welch test: F_(2, 2140.28)_ = 23.72, *p* < 0.0005). MDs (1.46 ± 2.98) reported higher numbers than nurses (1.13 ± 2.81) (*p* = 0.005, not reaching the adjusted significance threshold) and other staff (0.75 ± 1.94) (*p* < 0.0005). Nurses reported higher numbers than other staff (*p* = 0.0012). Chi-square tests showed differences in the distribution of several demographic variables (Supplementary Table 2). From particular importance, a higher proportion of females were detected in the nurses and other groups and more participants were tested positive for COVID-19 among MDs and in the nurse group.

In all questions with our 5-item Likert-scale, Kruskal–Wallis tests showed between-group differences. In general, nurses reached higher values on questions representative for subjective mental stress and an increased subjective burden (questions 9, 10, 12, 24, 25, 26, 27, 29, or 30) compared to both other groups. Nurses reached lower values on questions regarding the agreement with information policies, experienced support and preparation of the hospital regarding the COVID-19 pandemic (questions 11, 13, 14, 19, 20, 21, and 22). Overall, MDs reported lower values on stress- and subjective burden-related questions and achieved higher agreement with regard to structural measures and information policies. Even though we found significant group differences for all questions, the descriptive data (means, medians) showed that these differences were mostly subtle. Please see Table 3 for descriptive data and statistics of the three-group comparisons. As the proportion of females was higher among nurses and other groups compared to MDs, we performed all analyses separately for men and women (see Supplementary Tables 3 and 4) with subsequently mainly confirming the findings from the whole group.

**Table 3:** Results of the three-group comparisons (doctors vs. nurses vs. other staff) for all 5-item Likert-scale questions

	MD				Nurses				Others				Kruskal–Wallis			Mann–Whitney *U*	
	*N*	M	SD	Mdn	*N*	M	SD	Mdn	*N*	M	SD	Mdn	*H*	df	*p*		*P*
The COVID-19 pandemic has led to an increase in my daily workload (Q9)	1609	2.48	1.36	2	1310	3.60	1.17	4	733	3.02	1.25	3	477.1	2	< .0005^***^	MD vs. Nurses	< .0005^***^
																MD vs. Others	< .0005^***^
																Nurses vs. Others	< .0005^***^
Due to the COVID-19 pandemic I feel mentally strained (Q10)	1607	3.12	1.26	3	1299	3.63	1.16	4	728	3.53	1.10	4	133.9	2	< .0005^***^	MD vs. Nurses	< .0005^***^
																MD vs. Others	< .0005^***^
																Nurses vs. Others	.023
My superiors/my employer informed me sufficiently about COVID-19 (Q11)	1589	3.76	1.17	4	1300	3.56	1.18	4	726	3.71	1.18	4	24.4	2	< .0005^***^	MD vs. Nurses	< .0005^***^
																MD vs. Others	.260
																Nurses vs. Others	.004
Since the outbreak of the COVID-19 pandemic, the satisfaction with my job has worsened (Q12)	1596	3.13	1.34	3	1295	3.33	1.27	4	721	3.12	1.34	3	18.5	2	< .0005^***^	MD vs. Nurses	< .0005^***^
																MD vs. Others	.918
																Nurses vs Others	.001
I feel left alone by my employer (Q13)	1579	2.18	1.19	2	1295	2.67	1.25	3	723	2.38	1.20	2	121.6	2	< .0005^***^	MD vs. Nurses	< .0005^***^
																MD vs. Others	< .0005^***^
																Nurses vs. Others	< .0005^***^
I feel left alone by the responsible political decision-makers (Q14)	1591	2.57	1.26	2	1282	3.24	1.30	3	717	2.42	1.14	2	253.0	2	< .0005^***^	MD vs. Nurses	< .0005^***^
																MD vs. Others	.030
																Nurses vs. Others	< .0005^***^
The measures taken by the hospital administration have been appropriate (in terms of supply with information, protective equipment, and organization of work processes) (Q19)	1570	3.48	1.24	4	1273	3.01	1.25	3	720	3.28	1.20	4	105.7	2	< .0005^***^	MD vs. Nurses	< .0005^***^
																MD vs. Others	< .0005^***^
																Nurses vs. Others	< .0005^***^
In my opinion, the communication related to COVID-19 that came from the management of the hospital has been appropriate (Q20)	1554	3.69	1.18	4	1247	3.43	1.19	4	713	3.55	1.20	4	37.8	2	< .0005^***^	MD vs. Nurses	< .0005^***^
																MD vs. Others	.009
																Nurses vs. Others	.017
I have the impression that my efforts at work during the COVID-19 pandemic are being appreciated by the management of the hospital (Q21).	1546	3.39	1.20	3	1261	2.96	1.21	3	712	3.22	1.10	3	90.7	2	< .0005^***^	MD vs. Nurses	< .0005^***^
																MD vs. Others	< .0005^***^
																Nurses vs. Others	< .0005^***^
My hospital was/is well prepared with regard to the COVID-19 pandemic (Q22)	1559	3.65	1.16	4	1259	3.24	1.20	3	708	3.48	1.17	4	86.3	2	< .0005^***^	MD vs. Nurses	< .0005^***^
																MD vs. Others	.0005^**^
																Nurses vs. Others	< .0005^***^
Due to the COVID-19 pandemic, I have significantly less time for my personal life (Q24)	1566	2.10	1.23	2	1252	2.68	1.33	3	708	1.98	1.14	2	189.4	2	< .0005^***^	MD vs. Nurses	< .0005^***^
																MD vs. Others	.054
																Nurses vs. Others	< .0005^***^
My daily life has become more stressful due to the COVID-19 pandemic (Q25)	1563	2.81	1.40	3	1246	3.25	1.36	3	708	2.91	1.40	3	69.6	2	< .0005^***^	MD vs. Nurses	< .0005^***^
																MD vs. Others	.122
																Nurses vs. Others	< .0005^***^
Due to the COVID-19 pandemic, I am worrying more often about the future (Q26)	1564	3.17	1.27	3	1251	3.49	1.27	4	710	3.38	1.20	4	46.1	2	< .0005^***^	MD vs. Nurses	< .0005^***^
																MD vs. Others	< .0005^***^
																Nurses vs. Others	.033
Due to the COVID-19 pandemic, I am worrying more often about the well-being of my family (Q27)	1562	3.66	1.17	4	1249	4.14	1.05	4	708	3.88	1.13	4	151.2	2	< .0005^***^	MD vs. Nurses	< .0005^***^
																MD vs. Others	< .0005^***^
																Nurses vs. Others	< .0005^***^
I am afraid of catching the coronavirus myself (Q28)	1553	2.55	1.20	2	1241	2.84	1.28	3	708	2.61	1.19	2	36.9	2	< .0005^***^	MD vs. Nurses	< .0005^***^
																MD vs. Others	.256
																Nurses vs. Others	< .0005^***^
I fear that due to my daily exposure with it at work, I could pass on the coronavirus to my friends or relatives (Q29)	1557	3.30	1.31	4	1239	3.82	1.24	4	705	3.58	1.25	4	119.8	2	< .0005^***^	MD vs. Nurses	< .0005^***^
																MD vs. Others	< .0005^***^
																Nurses vs. Others	< .0005^***^
Since the COVID-19 pandemic, I have been sleeping less well (Q30)	1560	2.15	1.29	2	1247	2.61	1.38	2	707	2.34	1.31	2	86.2	2	< .0005^***^	MD vs. Nurses	< .0005^***^
																MD vs. Others	< .0005^***^
																Nurses vs. Others	< .0005^***^
In my setting, patients not infected with COVID-19 are adequately taken care of despite the COVID-19 pandemic (Q31)	1551	3.22	1.34	3	1191	3.39	1.22	4	684	3.08	1.31	3	24.6	2	< .0005^***^	MD vs. Nurses	.0023
																MD vs. Others	.016
																Nurses vs. Others	< .0005^***^
In my hospital setting, COVID-19-positive patients are adequately taken care of (Q32)	1458	3.81	1.12	4	1056	3.30	1.19	3	547	3.20	1.23	3	180.5	2	< .0005^***^	MD vs. Nurses	< .0005^***^
																MD vs. Others	< .0005^***^
																Nurses vs. Others	.204
I will continue to work in the healthcare area after the COVID-19 pandemic (Q33)	1543	4.78	0.63	5	1215	4.37	1.05	5	701	4.74	0.67	5	178.6	2	< .0005^***^	MD vs. Nurses	< .0005^***^
																MD vs. Others	.071
																Nurses vs. Others	< .0005^***^

Q: Question (see supplement for the complete questionnaire). ***p < .0005, **p < .002 (**p < .000667 in case of post hoc tests) (see Methods for the adjustment made to account for the multiple comparisons)

### Contrasting participants working on ICU/ER and COVID-19 wards to all others

Assuming that staff working on ICU, ER, and COVID-19 wards is exposed to a higher risk of being in contact with COVID-19 patients, we contrasted these groups together with all other participants. Those participants worked on average more hours overtime (3.36 ± 5.03 vs. 1.69 ± 3.74, Welch test: F_(1, 833.0)_ = 63.5, *p* < 0.0005), treated on average more patients with COVID-19 (8.48 ± 17.42 vs 1.82 ± 8.40, Welch test: F_(1, 750.6)_ = 93.6, *p* < 0.0005), and had on average more family members or friends who were COVID-19 positive (1.46 ± 3.56 vs 1.14 ± 2.52, Welch test: F_(1, 854.8)_ = 4.8, *p* = 0.029, trend). Participants working on ICU/ER/COVID-19 wards were more often COVID-19 positive (*p* < 0.0005), but belonged less often to the COVID-19 risk group (*p* = 0.013 (trend)). No differences in gender distribution between both groups (*p* = 0.205) were detected (for differences in other demographic variables and complete test statistics, see Supplementary Table 5). Overall, participants working in ICU/ER/COVID-19 wards reported higher rates of agreement with questions investigating stress and subjective burden and had lower rates of agreement with questions on structural measures and information policies. No group differences were detected with regard to subjective concerns about the future (question 26), to the fear to catch the virus (question 28), and to the question whether non-COVID-19 patients are adequately treated in the given setting (question 31). Higher rate of agreement was reported with regard to the adequate care of COVID-19 positive patients (question 32) among those participants working on ICU/ER/COVID-19 wards. Again, means and medians were in general comparable between groups (see Table 4).

**Table 4:** Results of the two-group comparisons (ER/ICU/COVID-19 wards vs. all other) for all 5-item Likert-scale questions

Item	ICU, ER, COVID-19 Ward				Others				*U*	*Z*	*p*
	*N*	Mdn	M	(SD)	*N*	*Mdn*	*M*	*(SD)*			
The COVID-19 pandemic has led to an increase in my daily workload (Q9)	704	4.00	3.46	(1.30)	2948	3.00	2.88	(1.36)	787137.0	-10.19	< .0005^***^
Due to the COVID-19 pandemic, I feel mentally strained (Q10)	699	4.00	3.54	(1.19)	2935	4.00	3.35	(1.22)	932714.5	-3.88	< .0005^***^
My superiors/my employer informed me sufficiently about COVID-19 (Q11)	700	4.00	3.51	(1.18)	2915	4.00	3.72	(1.17)	913287.5	-4.47	< .0005^***^
Since the outbreak of the COVID-19 pandemic, the satisfaction with my job has worsened (Q12)	695	3.00	3.29	(1.33)	2917	3.00	3.18	(1.31)	965374.0	-2.00	.045
I feel left alone by my employer (Q13)	696	2.00	2.63	(1.29)	2901	2.00	2.34	(1.22)	883087.5	-5.31	< .0005^***^
I feel left alone by the responsible political decision-makers (Q14)	691	3.00	3.21	(1.34)	2899	2.00	2.68	(1.27)	778983.0	-9.33	< .0005^***^
The measures taken by the hospital administration have been appropriate (in terms of supply with information, protective equipment, and organization of work processes) (Q19)	687	3.00	3.18	(1.26)	2876	4.00	3.30	(1.25)	935475.5	-2.24	.025
In my opinion, the communication related to COVID-19 that came from the management of the hospital has been appropriate (Q20)	674	4.00	3.37	(1.22)	2840	4.00	3.62	(1.18)	845498.5	-4.88	< .0005^***^
I have the impression that my efforts at work during the COVID-19 pandemic are being appreciated by the management of the hospital (Q21)	681	3.00	3.00	(1.24)	2838	3.00	3.25	(1.18)	858496.0	-4.66	< .0005^***^
My hospital was/is well prepared with regard to the COVID-19 pandemic (Q22)	682	4.00	3.35	(1.21)	2844	4.00	3.50	(1.18)	904964.0	-2.81	.005^**^
Due to the COVID-19 pandemic, I have significantly less time for my personal life (Q24)	683	3.00	2.73	(1.37)	2843	2.00	2.17	(1.24)	745744.0	-9.80	< .0005^***^
My daily life has become more stressful due to the COVID-19 pandemic (Q25)	681	3.00	3.10	(1.38)	2836	3.00	2.96	(1.40)	907134.0	-2.52	.012
Due to the COVID-19 pandemic, I am worrying more often about the future (Q26)	681	4.00	3.36	(1.28)	2844	4.00	3.32	(1.26)	947706.0	-0.90	.371
Due to the COVID-19 pandemic, I am worrying more often about the well-being of my family (Q27)	682	4.00	4.01	(1.12)	2837	4.00	3.84	(1.14)	871786.5	-4.24	< .0005^***^
I am afraid of catching the coronavirus myself (Q28)	676	3.00	2.72	(1.28)	2826	2.00	2.65	(1.22)	928046.0	-1.18	.237
I fear that due to my daily exposure with it at work, I could pass on the coronavirus to my friends or relatives (Q29)	678	4.00	3.81	(1.26)	2823	4.00	3.48	(1.29)	806745.5	-6.57	< .0005^***^
Since the COVID-19 pandemic, I have been sleeping less well (Q30)	678	2.00	2.61	(1.44)	2836	2.00	2.29	(1.32)	845699.0	-5.07	< .0005^***^
In my setting, patients not infected with COVID-19 are adequately taken care of despite the COVID-19 pandemic (Q31)	658	4.00	3.34	(1.28)	2768	3.00	3.23	(1.30)	869402.0	-1.86	.063
In my hospital setting, COVID-19-positive patients are adequately taken care of (Q32)	637	4.00	3.72	(1.13)	2424	4.00	3.48	(1.21)	684215.5	-4.57	< .0005^***^
I will continue to work in the healthcare area after the COVID-19 pandemic (Q33)	668	5.00	4.43	(1.00)	2791	5.00	4.68	(0.78)	814067.0	-7.03	< .0005^***^

Q: Question (see supplement for the complete questionnaire). ***p < 0.0005, **p < 0.002 (see Methods for the adjustment made to account for the multiple comparisons)

## Discussion

This study presents the first assessment regarding the subjective burden and views of German HCWs during the COVID-19 pandemic. We were able to show an increased level of subjective stress and concerns about the future and families as well as an increase of the subjective workload. Interestingly, the majority of participants reported no substantial impact of the COVID-19 pandemic on subjective sleep quality. Recent publications from Italy and China also showed increased levels of self-reported stress during the COVID-19 pandemic [4, 8]. In contrast to our findings, those publications also indicate high levels of insomnia possibly pointing towards the hypothesis that HCWs in Germany experienced less severe mental stress during the COVID-19 pandemic compared to other countries.

Against our expectations and in principle in contrast to international media reports, the general response patterns indicate in general positive evaluations of the communication policies, the level of support and protection, and the quality of care for patients with and without a COVID-19 infection. The availability of personal protective equipment (PPE) for HCWs who face an increased risk of infection during the COVID-19 pandemic is an ongoing and tremendously debated issue [11, 12] and in many countries the lack of PPE has been defined as a source of anxiety among HCWs (e.g., US media reports [13, 14]). However, German HCWs rated the overall availability of e.g. PPE as positive (question 19).

2.8% of our participants reported to have been tested positive for COVID-19 with even higher rates for doctors and nurses compared to other staff. Based on the infection numbers in Germany on May 1st (end of our survey), this would mean that our participants self-reported a more than tenfold increased risk to be COVID-19 positive compared to the general population (163009 COVID-19-positive persons in Germany [15]/83149300 inhabitants in Germany [16]; 0.2%). This self-reported infection rate is higher than, e.g., among HCWs in China (Wuhan) [17] or The Netherlands [18], were rates ~ 1% were reported on the basis of performed COVID-19 tests [19]. Whether this finding is the consequence of an increased test frequency in Germany, whether our self-reporting data overestimate the infection rates or whether HCWs in Germany have an increased risk to be infected by COVID-19 remains elusive and cannot be answered with our study design.

In general, nurses reported higher levels of stress and subjective burden and lower levels of work satisfaction and experienced support compared to MDs and—to a certain extent—compared to other HCWs. One could assume that nurses are at a higher risk to experience psychosocial stress than MDs during the COVID-19 pandemic. This might be due to the fact that nurses might spend a longer period of time in direct patient contact (including COVID-19 patients) and, thus, might be more exposed not only to the concerns and fears of the patients and their relatives during the pandemic, but also to the virus itself. Despite the fact that no relevant differences in response patterns between males and females were detected, gender differences should be acknowledged when comparing nurses with doctors. One recent meta-analysis of 13 studies (12 studies conducted in China and one in Singapore) comprising a total of 33062 participants showed that female HCWs and nurses might exhibit higher rates of negative affective symptoms during the COVID-19 outbreak [20]. Our findings are in line with the meta-analytic evidence, but show for the first time this relationship in a large-scale database from a European perspective. Irrespective of the COVID-19 pandemic, nurses, especially those working in inpatient settings, are presumed to experience a high amount of stress and report reduced satisfaction with their jobs [21]. The higher level of psychological distress among nurses highlights the need to provide sufficient support specifically for this group to reduce their risk to develop stress-related disorders now and in the future. Higher agreement rates with regard to our stress-related questions were also identified for those HCWs working on ER/ICU/COVID-19 special wards and this group reported an infection rate of 4.8%. As for nurses, our data indicate that this group may have an increased risk to develop stress-related disorders and must receive special support. Factors that may increase the stress particular in this group include the lack of PPE, concerns regarding the individual future, the feeling to have less control of the situation, the safety of the individual families, and the risk to catch the virus in the respective special working environment [22]. However, despite these higher agreement rates, only a few questions reached significance level (see Table 4) indicating a high level of professional and personal competence. In this context, one must acknowledge that the COVID-19 outbreak in Germany was less dramatic than in the other European countries. Finally, the values for increased working hours must be interpreted within caution taking into account the German perspective. All hospitals had to stop all routine procedures and operations following governmental resolutions and some hospitals to be prepared for COVID-19 patients. Thus, many HCWs did not report an increase in working hours as many hospital beds were empty during the study period. As expected, staff working on ICU, ER, and COVID-19 reported more hours overtime than all other participants and MDs and nurses more hours overtime than other HCWs during the study period.

The present study has some limitations. First, online surveys have specific disadvantages such as not being representative, not providing comprehensive information regarding the participants, or the risk to receive fake answers. Second, we did not use validated questionnaires. Third, we had no control group of non-HCWs. Assuming that the COVID-19 pandemic has an impact on the well-being of all people, the specificity of our findings needs to be set into the societal perspective. However, we calculated contrasts between different groups of HCWs and were able to show meaningful differences between our different study groups. Moreover, our findings are not controlled for pre-existing mental conditions and otherwise adverse individual life circumstances (e.g., substance abuse or financial worries) highlighting the need to confirm our findings in future studies using direct interviews. Next, the lacking longitudinal design poses a further limitation as we cannot evaluate whether those HCWs with high rankings for subjective psychological burden or those feeling themselves abandoned are at an increased risk to develop stress-related disorders. The advantage of our survey is that we were able to receive answers from a large cohort of HCWs, that our sample size exceeds the sample size of other international surveys conducted during the COVID-19 or other pandemics, and that we were able to show profession-specific differences in mental-health burden.

To conclude, this is the first assessment of subjective burden and views among German HCWs during the COVID-19 pandemic. Our results are in line with the recent findings from China and Italy showing an increased subjective burden of HCWs. However, our survey extends the previous reports by confirming that especially nurses and professionals working in a high-risk environment to catch the COVID-19 virus experience higher levels of subjective burden than others. Moreover, our survey shows that German HCWs’ rate implemented structural measures and communication strategies as positive. Whether the latter is related to the well-equipped health-care system or to the different dynamics of virus spread in Germany compared to other countries needs to be answered in future studies.

## Electronic supplementary material

Below is the link to the electronic supplementary material.

Supplementary material 1 (DOCX 88 kb)

## Data Availability

Data can be requested from the corresponding author.
