# Supplementary material for: Subjective burden and perspectives of German healthcare workers during the COVID-19 pandemic
Source: Eur Arch Psychiatry Clin Neurosci. 2020 Aug 19;271(2):271–81. doi: 10.1007/s00406-020-01183-2 (PMC7437642; doi:10.1007/s00406-020-01183-2)
Supplement: Supplementary file 1 — Supplementary material 1 (DOCX 88 kb) [file 406_2020_1183_MOESM1_ESM.docx]

| **Item** |  |
| --- | --- |
|  | ***N* (%)** |
| Total  Anesthesiology  Opthalmology  Surgery  Dermatology  Obsterics and Gynecology  ENT  Internal Medicine  Child and Adolescent Psychiatry  Pediatrics  Laboratory Medicine  Micorbiology  Neurology  Nuclear Medicine  Psychiatry and Psychotherapy  Psychosomatic Medicine  Radiology  Other  Radiation Medicine  Urology | 3644 (99.3)  125 (3.4)  29 (0.8)  1040 (28.5)  34 (0.9)  65 (1.8)  14 (0.4)  312 (8.6)  76 (2.1)  159 (4.4)  1 (0.03)  1 (0.03)  100 (2.7)  2 (0.1)  1370 (37.6)  84 (2.3)  12 (0.3)  172 (4.7)  34 (0.9)  14 (0.4) |

**Supplementary Table 1:** Distribution of working fields

|  | **MD** | | **Nurses** | | **Others** | |  |  |
| --- | --- | --- | --- | --- | --- | --- | --- | --- |
|  | ***N*** | **(%)** | ***N*** | **(%)** | ***N*** | **(%)** | **χ^2^ (*df)*** | ***P*** |
| **Gender** | | | | | | | | |
| Female | 690 | (42.9%) | 931 | (71.4%) | 604 | (82.6%) |  |  |
| Male | 919 | (57.1%) | 370 | (28.4%) | 126 | (17.2%) | 430.06 (4) | <.0005*** |
| Third | 1 | (0.1%) | 3 | (0.2%) | 1 | (0.1%) |  |  |
| **Age (in years)** |  |  |  |  |  |  |  |  |
| 18-30 | 161 | (10.0%) | 349 | (26.6%) | 189 | (25.7%) |  |  |
| 31-40 | 437 | (27.1%) | 314 | (24.0%) | 212 | (28.8%) | 183.60 (8) | <.0005*** |
| 41-50 | 352 | (21.8%) | 272 | (20.7%) | 133 | (18.1%) |  |  |
| 51-60 | 478 | (29.6%) | 293 | (22.3%) | 154 | (21.0%) |  |  |
| >60 | 185 | (11.5%) | 83 | (6.3%) | 47 | (6.4%) |  |  |
| **Unit/Ward** | | | | | | | | |
| Ambulatory | 303 | (18.7%) | 136 | (10.3%) | 162 | (22.0%) |  |  |
| Emergency Room (ER) | 96 | (5.9%) | 80 | (6.1%) | 12 | (1.6%) |  |  |
| Unit Floor / Ward | 1076 | (66.5%) | 769 | (58.4%) | 517 | (70.3%) | 266.52 (8) | <.0005*** |
| COVID-19 ward | 40 | (2.5%) | 79 | (6.0%) | 10 | (1.4%) |  |  |
| Intensive Care Unit (ICU) | 102 | (6.3%) | 253 | (19.2%) | 34 | (4.6%) |  |  |
| **Type of hospital** | | | | | | | | |
| Other | 1114 | (69.5%) | 617 | (47.4%) | 437 | (61.4%) | 145.90 (2) | <.0005*** |
| University Hospital | 490 | (30.5%) | 684 | (52.6%) | 275 | (38.6%) |  |  |
| **COVID-19 risk group** | | | | | | | | |
| Yes | 404 | (25.8%) | 386 | (30.8%) | 148 | (20.7%) | 24.55 (2) | <.0005*** |
| No | 1162 | (74.2%) | 869 | (69.2%) | 568 | (79.3%) |  |  |
| **Positive COVID-19 Test** | | | | | | | | |
| Yes | 57 | (3.6%) | 39 | (3.1%) | 4 | (0.6%) | 17.39 (2) | <.0005*** |
| No | 1521 | (96.4%) | 1222 | (96.9%) | 713 | (99.4%) |  |  |

**Supplementary Table 2:** Demographic characteristics for the 3-group comparison (MD vs. nurses vs. others), *** p < 0.0005

|  | **MD** | | | | **Nurses** | | | | **Others** | | | | **Kruskal-Wallis** | | | **Mann-Whitney-U** | |
| --- | --- | --- | --- | --- | --- | --- | --- | --- | --- | --- | --- | --- | --- | --- | --- | --- | --- |
|  | *N* | *M* | *SD* | *Mdn* | *N* | *M* | *SD* | *Mdn* | *N* | *M* | *SD* | *Mdn* | *H* | *df* | *P* |  | *p* |
| The COVID-19 pandemic has led to an increase in my daily workload. | 687 | 2.56 | 1.36 | 2 | 925 | 3.56 | 1.16 | 4 | 603 | 3.02 | 1.27 | 3 | 217.6 | 2 | <.0005^***^ | MD vs. Nurses | <.0005^***^ |
|  |  |  |  |  |  |  |  |  |  |  |  |  |  |  |  | MD vs. Others | <.0005^***^ |
|  |  |  |  |  |  |  |  |  |  |  |  |  |  |  |  | Nurses vs. Others | <.0005^***^ |
| Due to the COVID-19 pandemic I feel mentally strained. | 684 | 3.36 | 1.19 | 4 | 922 | 3.66 | 1.13 | 4 | 599 | 3.59 | 1.09 | 4 | 26.8 | 2 | <.0005^***^ | MD vs. Nurses | <.0005^***^ |
|  |  |  |  |  |  |  |  |  |  |  |  |  |  |  |  | MD vs. Others | .0005^**^ |
|  |  |  |  |  |  |  |  |  |  |  |  |  |  |  |  | Nurses vs. Others | .181 |
| My superiors/my employer informed me sufficiently about COVID-19. | 682 | 3.68 | 1.16 | 4 | 919 | 3.65 | 1.15 | 4 | 596 | 3.73 | 1.16 | 4 | 2.9 | 2 | .232 |  |  |
|  |  |  |  |  |  |  |  |  |  |  |  |  |  |  |  | n.a. |  |
|  |  |  |  |  |  |  |  |  |  |  |  |  |  |  |  |  |  |
| Since the outbreak of the COVID-19 pandemic, the satisfaction with my job has worsened. | 684 | 3.16 | 1.32 | 3 | 918 | 3.31 | 1.27 | 3 | 592 | 3.13 | 1.34 | 3 | 7.2 | 2 | .027 |  |  |
|  |  |  |  |  |  |  |  |  |  |  |  |  |  |  |  | n.a. |  |
|  |  |  |  |  |  |  |  |  |  |  |  |  |  |  |  |  |  |
| I feel left alone by my employer. | 677 | 2.26 | 1.20 | 2 | 919 | 2.63 | 1.26 | 3 | 593 | 2.38 | 1.19 | 2 | 38.8 | 2 | <.0005^***^ | MD vs. Nurses | <.0005^***^ |
|  |  |  |  |  |  |  |  |  |  |  |  |  |  |  |  | MD vs. Others | .056 |
|  |  |  |  |  |  |  |  |  |  |  |  |  |  |  |  | Nurses vs. Others | <.0005^***^ |
| I feel left alone by the responsible political decision-makers. | 682 | 2.57 | 1.22 | 2 | 906 | 3.24 | 1.29 | 3 | 588 | 2.41 | 1.13 | 2 | 178.2 | 2 | <.0005^***^ | MD vs. Nurses | <.0005^***^ |
|  |  |  |  |  |  |  |  |  |  |  |  |  |  |  |  | MD vs. Others | .038 |
|  |  |  |  |  |  |  |  |  |  |  |  |  |  |  |  | Nurses vs. Others | <.0005^***^ |
| The measures taken by the hospital administration have been appropriate (in terms of supply with information, protective equipment, organization of work processes). | 677 | 3.35 | 1.22 | 4 | 905 | 3.04 | 1.25 | 3 | 591 | 3.28 | 1.19 | 4 | 26.8 | 2 | <.0005^***^ | MD vs. Nurses | <.0005^***^ |
|  |  |  |  |  |  |  |  |  |  |  |  |  |  |  |  | MD vs. Others | .265 |
|  |  |  |  |  |  |  |  |  |  |  |  |  |  |  |  | Nurses vs. Others | <.0005^***^ |
| In my opinion, the communication related to COVID-19 that came from the management of the hospital has been appropriate. | 672 | 3.58 | 1.22 | 4 | 887 | 3.45 | 1.17 | 4 | 586 | 3.58 | 1.21 | 4 | 8.2 | 2 | .017 |  |  |
|  |  |  |  |  |  |  |  |  |  |  |  |  |  |  |  | n.a. |  |
|  |  |  |  |  |  |  |  |  |  |  |  |  |  |  |  |  |  |
| I have the impression that my efforts at work during the Covid-19 pandemic are being appreciated by the management of the hospital. | 668 | 3.30 | 1.21 | 3 | 894 | 2.98 | 1.21 | 3 | 584 | 3.24 | 1.08 | 3 | 32.8 | 2 | <.0005^***^ | MD vs. Nurses | <.0005^***^ |
|  |  |  |  |  |  |  |  |  |  |  |  |  |  |  |  | MD vs. Others | .206 |
|  |  |  |  |  |  |  |  |  |  |  |  |  |  |  |  | Nurses vs. Others | <.0005^***^ |
| My hospital was/is well prepared with regard to the Covid-19 pandemic. | 671 | 3.56 | 1.14 | 4 | 895 | 3.36 | 1.17 | 4 | 582 | 3.49 | 1.16 | 4 | 12.8 | 2 | .0017^**^ | MD vs. Nurses | .001 |
|  |  |  |  |  |  |  |  |  |  |  |  |  |  |  |  | MD vs. Others | .341 |
|  |  |  |  |  |  |  |  |  |  |  |  |  |  |  |  | Nurses vs. Others | .022 |
| Due to the COVID-19 pandemic, I have significantly less time for my personal life. | 675 | 2.17 | 1.23 | 2 | 886 | 2.65 | 1.32 | 3 | 582 | 1.98 | 1.13 | 2 | 108.7 | 2 | <.0005^***^ | MD vs. Nurses | <.0005^***^ |
|  |  |  |  |  |  |  |  |  |  |  |  |  |  |  |  | MD vs. Others | .012 |
|  |  |  |  |  |  |  |  |  |  |  |  |  |  |  |  | Nurses vs. Others | <.0005^***^ |
| My daily life has become more stressful due to the COVID-19 pandemic. | 672 | 2.97 | 1.37 | 3 | 883 | 3.20 | 1.38 | 3 | 581 | 2.98 | 1.42 | 3 | 13.8 | 2 | .001^**^ | MD vs. Nurses | .001 |
|  |  |  |  |  |  |  |  |  |  |  |  |  |  |  |  | MD vs. Others | .862 |
|  |  |  |  |  |  |  |  |  |  |  |  |  |  |  |  | Nurses vs. Others | .004 |
| Due to the COVID-19 pandemic, I am worrying more often about the future. | 671 | 3.26 | 1.23 | 4 | 888 | 3.60 | 1.23 | 4 | 582 | 3.42 | 1.19 | 4 | 31.6 | 2 | <.0005^***^ | MD vs. Nurses | <.0005^***^ |
|  |  |  |  |  |  |  |  |  |  |  |  |  |  |  |  | MD vs. Others | .024 |
|  |  |  |  |  |  |  |  |  |  |  |  |  |  |  |  | Nurses vs. Others | .002 |
| Due to the COVID-19 pandemic I am worrying more often about the well-being of my family. | 673 | 3.89 | 1.08 | 4 | 887 | 4.28 | 0.96 | 5 | 581 | 3.96 | 1.07 | 4 | 78.7 | 2 | <.0005^***^ | MD vs. Nurses | <.0005^***^ |
|  |  |  |  |  |  |  |  |  |  |  |  |  |  |  |  | MD vs. Others | .137 |
|  |  |  |  |  |  |  |  |  |  |  |  |  |  |  |  | Nurses vs. Others | <.0005^***^ |
| I am afraid of catching the Coronavirus myself. | 669 | 2.61 | 1.19 | 2 | 882 | 2.87 | 1.27 | 3 | 581 | 2.64 | 1.21 | 2 | 19.6 | 2 | <.0005^***^ | MD vs. Nurses | <.0005^***^ |
|  |  |  |  |  |  |  |  |  |  |  |  |  |  |  |  | MD vs. Others | .766 |
|  |  |  |  |  |  |  |  |  |  |  |  |  |  |  |  | Nurses vs. Others | .0005^**^ |
| I fear that due to my daily exposure with it at work, I could pass on the coronavirus to my friends or relatives. | 668 | 3.59 | 1.24 | 4 | 879 | 3.92 | 1.21 | 4 | 580 | 3.64 | 1.24 | 4 | 38.9 | 2 | <.0005^***^ | MD vs. Nurses | <.0005^***^ |
|  |  |  |  |  |  |  |  |  |  |  |  |  |  |  |  | MD vs. Others | .492 |
|  |  |  |  |  |  |  |  |  |  |  |  |  |  |  |  | Nurses vs. Others | <.0005^***^ |
| Since the COVID-19 pandemic, I have been sleeping less well. | 671 | 2.34 | 1.36 | 2 | 884 | 2.69 | 1.40 | 3 | 580 | 2.42 | 1.34 | 2 | 27.8 | 2 | <.0005^***^ | MD vs. Nurses | <.0005^***^ |
|  |  |  |  |  |  |  |  |  |  |  |  |  |  |  |  | MD vs. Others | .212 |
|  |  |  |  |  |  |  |  |  |  |  |  |  |  |  |  | Nurses vs. Others | <.0005^***^ |
| In my setting, patients not infected with Covid-19 are adequately taken care of despite the Covid-19 pandemic. | 665 | 3.22 | 1.28 | 4 | 840 | 3.46 | 1.21 | 4 | 562 | 3.10 | 1.28 | 3 | 27.3 | 2 | <.0005^***^ | MD vs. Nurses | .0005^**^ |
|  |  |  |  |  |  |  |  |  |  |  |  |  |  |  |  | MD vs. Others | .115 |
|  |  |  |  |  |  |  |  |  |  |  |  |  |  |  |  | Nurses vs. Others | <.0005^***^ |
| In my hospital setting, COVID-19 positive patients are adequately taken care of. | 623 | 3.69 | 1.16 | 4 | 744 | 3.35 | 1.17 | 3 | 447 | 3.22 | 1.21 | 3 | 51.4 | 2 | <.0005^***^ | MD vs. Nurses | <.0005^***^ |
|  |  |  |  |  |  |  |  |  |  |  |  |  |  |  |  | MD vs. Others | <.0005^***^ |
|  |  |  |  |  |  |  |  |  |  |  |  |  |  |  |  | Nurses vs. Others | .106 |
| I will continue to work in the healthcare area after the COVID-19 pandemic. | 664 | 4.80 | 0.53 | 5 | 861 | 4.43 | 0.99 | 5 | 576 | 4.77 | 0.61 | 5 | 88.3 | 2 | <.0005^***^ | MD vs. Nurses | <.0005^***^ |
|  |  |  |  |  |  |  |  |  |  |  |  |  |  |  |  | MD vs. Others | .380 |
|  |  |  |  |  |  |  |  |  |  |  |  |  |  |  |  | Nurses vs. Others | <.0005^***^ |

**Supplementary Table 3:** Results of the 3-group comparisons (only females); ^***^ *P* < .0005; ^**^ *p* < .002 (^**^ *p* < .000667 in case of post hoc tests); n.a. = not applicable (omnibus test was not significant)

|  | **MD** | | | | **Nurses** | | | | **Others** | | | | **Kruskal-Wallis** | | | **Mann-Whitney-U** | |
| --- | --- | --- | --- | --- | --- | --- | --- | --- | --- | --- | --- | --- | --- | --- | --- | --- | --- |
|  | *N* | *M* | *SD* | *Mdn* | *N* | *M* | *SD* | *Mdn* | *N* | *M* | *SD* | *Mdn* | *H* | *df* | *p* |  | *p* |
| The COVID-19 pandemic has led to an increase in my daily workload. | 914 | 2.42 | 1.36 | 2 | 369 | 3.70 | 1.19 | 4 | 125 | 2.99 | 1.19 | 3 | 219.2 | 2 | <.0005^***^ | MD vs. Nurses | <.0005^***^ |
|  |  |  |  |  |  |  |  |  |  |  |  |  |  |  |  | MD vs. Others | <.0005^***^ |
|  |  |  |  |  |  |  |  |  |  |  |  |  |  |  |  | Nurses vs. Others | <.0005^***^ |
| Due to the COVID-19 pandemic I feel mentally strained. | 915 | 2.95 | 1.28 | 3 | 361 | 3.56 | 1.21 | 4 | 124 | 3.27 | 1.11 | 4 | 61.0 | 2 | <.0005^***^ | MD vs. Nurses | <.0005^***^ |
|  |  |  |  |  |  |  |  |  |  |  |  |  |  |  |  | MD vs. Others | .010 |
|  |  |  |  |  |  |  |  |  |  |  |  |  |  |  |  | Nurses vs. Others | .007 |
| My superiors/my employer informed me sufficiently about COVID-19. | 899 | 3.82 | 1.17 | 4 | 365 | 3.38 | 1.21 | 4 | 125 | 3.58 | 1.25 | 4 | 40.6 | 2 | <.0005^***^ | MD vs. Nurses | <.0005^***^ |
|  |  |  |  |  |  |  |  |  |  |  |  |  |  |  |  | MD vs. Others | .037 |
|  |  |  |  |  |  |  |  |  |  |  |  |  |  |  |  | Nurses vs. Others | .084 |
| Since the outbreak of the COVID-19 pandemic, the satisfaction with my job has worsened. | 904 | 3.11 | 1.35 | 3 | 361 | 3.39 | 1.26 | 4 | 124 | 3.03 | 1.35 | 3 | 12.4 | 2 | .0021 |  |  |
|  |  |  |  |  |  |  |  |  |  |  |  |  |  |  |  | n.a. |  |
|  |  |  |  |  |  |  |  |  |  |  |  |  |  |  |  |  |  |
| I feel left alone by my employer. | 894 | 2.11 | 1.19 | 2 | 360 | 2.76 | 1.24 | 3 | 125 | 2.37 | 1.23 | 2 | 75.5 | 2 | <.0005^***^ | MD vs. Nurses | <.0005^***^ |
|  |  |  |  |  |  |  |  |  |  |  |  |  |  |  |  | MD vs. Others | .018 |
|  |  |  |  |  |  |  |  |  |  |  |  |  |  |  |  | Nurses vs. Others | .002 |
| I feel left alone by the responsible political decision-makers. | 901 | 2.56 | 1.30 | 2 | 360 | 3.21 | 1.32 | 3 | 124 | 2.46 | 1.18 | 2 | 65.8 | 2 | <.0005^***^ | MD vs. Nurses | <.0005^***^ |
|  |  |  |  |  |  |  |  |  |  |  |  |  |  |  |  | MD vs. Others | .535 |
|  |  |  |  |  |  |  |  |  |  |  |  |  |  |  |  | Nurses vs. Others | <.0005^***^ |
| The measures taken by the hospital administration have been appropriate (in terms of supply with information, protective equipment, organization of work processes). | 885 | 3.59 | 1.24 | 4 | 352 | 2.93 | 1.23 | 3 | 124 | 3.33 | 1.21 | 4 | 72.5 | 2 | <.0005^***^ | MD vs. Nurses | <.0005^***^ |
|  |  |  |  |  |  |  |  |  |  |  |  |  |  |  |  | MD vs. Others | .015 |
|  |  |  |  |  |  |  |  |  |  |  |  |  |  |  |  | Nurses vs. Others | .002 |
| In my opinion, the communication related to COVID-19 that came from the management of the hospital has been appropriate. | 874 | 3.78 | 1.14 | 4 | 345 | 3.38 | 1.24 | 4 | 123 | 3.43 | 1.16 | 4 | 32.5 | 2 | <.0005^***^ | MD vs. Nurses | <.0005^***^ |
|  |  |  |  |  |  |  |  |  |  |  |  |  |  |  |  | MD vs. Others | .001 |
|  |  |  |  |  |  |  |  |  |  |  |  |  |  |  |  | Nurses vs. Others | .830 |
| I have the impression that my efforts at work during the COVID-19 pandemic are being appreciated by the management of the hospital. | 870 | 3.46 | 1.19 | 4 | 351 | 2.94 | 1.22 | 3 | 123 | 3.20 | 1.15 | 3 | 46.6 | 2 | <.0005^***^ | MD vs. Nurses | <.0005^***^ |
|  |  |  |  |  |  |  |  |  |  |  |  |  |  |  |  | MD vs. Others | .019 |
|  |  |  |  |  |  |  |  |  |  |  |  |  |  |  |  | Nurses vs. Others | .035 |
| My hospital was/is well prepared with regard to the COVID-19 pandemic. | 880 | 3.71 | 1.18 | 4 | 349 | 2.94 | 1.20 | 3 | 122 | 3.40 | 1.20 | 3 | 101.7 | 2 | <.0005^***^ | MD vs. Nurses | <.0005^***^ |
|  |  |  |  |  |  |  |  |  |  |  |  |  |  |  |  | MD vs. Others | .004 |
|  |  |  |  |  |  |  |  |  |  |  |  |  |  |  |  | Nurses vs. Others | .0005^**^ |
| Due to the COVID-19 pandemic, I have significantly less time for my personal life. | 883 | 2.05 | 1.22 | 2 | 350 | 2.73 | 1.36 | 3 | 121 | 1.94 | 1.21 | 1 | 73.4 | 2 | <.0005^***^ | MD vs. Nurses | <.0005^***^ |
|  |  |  |  |  |  |  |  |  |  |  |  |  |  |  |  | MD vs. Others | .266 |
|  |  |  |  |  |  |  |  |  |  |  |  |  |  |  |  | Nurses vs. Others | <.0005^***^ |
| My daily life has become more stressful due to the COVID-19 pandemic. | 883 | 2.69 | 1.40 | 3 | 347 | 3.34 | 1.29 | 4 | 123 | 2.54 | 1.28 | 2 | 60.5 | 2 | <.0005^***^ | MD vs. Nurses | <.0005^***^ |
|  |  |  |  |  |  |  |  |  |  |  |  |  |  |  |  | MD vs. Others | .331 |
|  |  |  |  |  |  |  |  |  |  |  |  |  |  |  |  | Nurses vs. Others | <.0005^***^ |
| Due to the COVID-19 pandemic, I am worrying more often about the future. | 885 | 3.11 | 1.30 | 3 | 347 | 3.19 | 1.31 | 3 | 123 | 3.19 | 1.23 | 4 | 1.1 | 2 | .568 |  |  |
|  |  |  |  |  |  |  |  |  |  |  |  |  |  |  |  | n.a. |  |
|  |  |  |  |  |  |  |  |  |  |  |  |  |  |  |  |  |  |
| Due to the COVID-19 pandemic I am worrying more often about the well-being of my family. | 882 | 3.49 | 1.21 | 4 | 346 | 3.79 | 1.18 | 4 | 122 | 3.48 | 1.27 | 4 | 19.1 | 2 | <.0005^***^ | MD vs. Nurses | <.0005^***^ |
|  |  |  |  |  |  |  |  |  |  |  |  |  |  |  |  | MD vs. Others | .959 |
|  |  |  |  |  |  |  |  |  |  |  |  |  |  |  |  | Nurses vs. Others | .014 |
| I am afraid of catching the Coronavirus myself. | 876 | 2.50 | 1.20 | 2 | 343 | 2.76 | 1.32 | 3 | 123 | 2.47 | 1.13 | 2 | 9.6 | 2 | .008 |  |  |
|  |  |  |  |  |  |  |  |  |  |  |  |  |  |  |  | n.a. |  |
|  |  |  |  |  |  |  |  |  |  |  |  |  |  |  |  |  |  |
| I fear that due to my daily exposure with it at work, I could pass on the coronavirus to my friends or relatives. | 881 | 3.08 | 1.32 | 3 | 344 | 3.57 | 1.31 | 4 | 121 | 3.33 | 1.25 | 4 | 36.3 | 2 | <.0005^***^ | MD vs. Nurses | <.0005^***^ |
|  |  |  |  |  |  |  |  |  |  |  |  |  |  |  |  | MD vs. Others | .054 |
|  |  |  |  |  |  |  |  |  |  |  |  |  |  |  |  | Nurses vs. Others | .042 |
| Since the COVID-19 pandemic, I have been sleeping less well. | 881 | 2.00 | 1.23 | 2 | 347 | 2.41 | 1.32 | 2 | 122 | 1.94 | 1.12 | 2 | 30.2 | 2 | <.0005^***^ | MD vs. Nurses | <.0005^***^ |
|  |  |  |  |  |  |  |  |  |  |  |  |  |  |  |  | MD vs. Others | .892 |
|  |  |  |  |  |  |  |  |  |  |  |  |  |  |  |  | Nurses vs. Others | .00061** |
| In my setting, patients not infected with COVID-19 are adequately taken care of despite the Covid-19 pandemic. | 878 | 3.22 | 1.38 | 3 | 337 | 3.24 | 1.22 | 3 | 118 | 2.97 | 1.41 | 3 | 3.7 | 2 | .156 |  |  |
|  |  |  |  |  |  |  |  |  |  |  |  |  |  |  |  | n.a. |  |
|  |  |  |  |  |  |  |  |  |  |  |  |  |  |  |  |  |  |
| In my hospital setting, COVID-19 positive patients are adequately taken care of. | 828 | 3.91 | 1.09 | 4 | 298 | 3.18 | 1.21 | 3 | 97 | 3.09 | 1.30 | 3 | 109.0 | 2 | <.0005^***^ | MD vs. Nurses | <.0005^***^ |
|  |  |  |  |  |  |  |  |  |  |  |  |  |  |  |  | MD vs. Others | <.0005^***^ |
|  |  |  |  |  |  |  |  |  |  |  |  |  |  |  |  | Nurses vs. Others | .587 |
| I will continue to work in the healthcare area after the COVID-19 pandemic. | 871 | 4.77 | 0.70 | 5 | 339 | 4.26 | 1.16 | 5 | 120 | 4.65 | 0.86 | 5 | 88.3 | 2 | <.0005^***^ | MD vs. Nurses | <.0005^***^ |
|  |  |  |  |  |  |  |  |  |  |  |  |  |  |  |  | MD vs. Others | .090 |
|  |  |  |  |  |  |  |  |  |  |  |  |  |  |  |  | Nurses vs. Others | <.0005^***^ |
| **Supplementary Table 4:** Results of the 3-group comparisons (only males); ^***^ *p* < .0005; ^**^ *p* < .002 (^**^ *p* < .000667 in case of post hoc tests); n.a. = not applicable (omnibus test was not significant) | | | | | | | | | | | | | | | | | |

|  | **ER, ICU, COVID-19** | | **Others** | |  |  |
| --- | --- | --- | --- | --- | --- | --- |
|  | ***N*** | **(%)** | ***N*** | **(%)** | **χ^2^ (*df)*** | ***p*** |
| **Gender** | | | | | | |
| Male | 257 | (36.6%) | 1158 | (39.4%) |  |  |
| Female | 444 | (63.2%) | 1781 | (60.5%) | 3.17 (2) | .205 |
| Third | 2 | (0.3%) | 3 | (0.1%) |  |  |
| **Age (in years)** |  |  |  |  |  |  |
| 18-30 | 190 | (27.0%) | 509 | (17.2%) |  |  |
| 31-40 | 228 | (32.4%) | 735 | (24.9%) | 86.95 (4) | <.0005 *** |
| 41-50 | 137 | (19.5%) | 620 | (21.0%) |  |  |
| 51-60 | 121 | (17.2%) | 804 | (27.2%) |  |  |
| >60 | 27 | (3.8%) | 288 | (9.7%) |  |  |
| **Unit/Ward** | | | | | | |
| Ambulatory | 0 | (0%) | 601 | (20.3%) |  |  |
| Emergency Room (ER) | 188 | (26.6%) | 0 | (0%) |  |  |
| Unit Floor / Ward | 0 | (0%) | 2362 | (79.7%) | 3669.00 (4) | <.0005 *** |
| COVID-19 ward | 129 | (18.3%) | 0 | (0%) |  |  |
| Intensive Care Unit (ICU) | 389 | (55.1%) | 0 | (0%) |  |  |
| **Type of hospital** | | | | | | |
| Other | 314 | (44.7%) | 1854 | (63.6%) | 83.92 (1) | <.0005 *** |
| University Hospital | 388 | (55.3%) | 1061 | (36.4%) |  |  |
| **COVID-19 risk group** | | | | | | |
| Yes | 154 | (22.7%) | 784 | (27.4%) | 6.11 (1) | .013 |
| No | 523 | (77.3%) | 2076 | (72.6%) |  |  |
| **Positive COVID-19 Test** | | | | | | |
| Yes | 33 | (4.8%) | 67 | (2.3%) | 12.61 (1) | <.0005 *** |
| No | 650 | (95.2%) | 2806 | (97.7%) |  |  |

**Supplementary Table 5:** Demographic characteristics for the 2-group comparison (ER/ICU/COVID-19 wards vs. others), *** p < 0.0005.

**Questionnaire:** This questionnaire was provided in German language and translated for this publication.

| **Introduction** |  | |
| --- | --- | --- |
| Dear ladies and gentlemen and fellow colleagues,    We would like to circulate a questionnaire among doctors, nurses and other healthcare professionals in various hospitals and subspecialties to investigate the amount of subjective exposure at the respective health care workplaces, as well as the degrees of anxiety and preoccupation related to the COVID-19 pandemic. The aim of this research project is to foster the development of preventive strategies and decision-making protocols that might be available for health care workers in similar situations in the future. This questionnaire is part of a research project between the medical faculty of the University of Augsburg and the University Hospital, LMU Munich. The content of the questionnaire was a priori approved by the German Society for Psychiatry and Psychotherapy, Psychosomatic and Neurology (www.dgppn.de).  Employees from hospitals throughout the entire federal territory are invited to participate in a 10 min survey that will contribute to a better understanding of subsequent psychological effects of COVID-19 on our national health care system.  Thank you for your time and support.  Sincerely yours,  Prof. Dr. Alkomiet Hasan  Department of Psychiatry and Psychotherapy, University of Augsburg  Prof. Dr. Miriam Kunz  Department of Medical Psychology and Sociology, University of Augsburg  Prof. Dr. Peter Falkai  Department of Psychiatry and Psychotherapy, LMU Munich | | |
| **Notice of data usage**: | |  |
| No personal information will be registered and please refrain from submitting personal data or data that could be related to you personally. An anonymous questionnaire has been configurated in the settings of this survey. As a consequence, the database of this survey does not register neither who participated in the survey and submitted responses, nor when and from where. In the event that personal data or data that could be related to you as a person is submitted, this information will be treated confidentially. The submission of personal data is voluntary. There is no transfer of data to a third party. Having received your data, we will delete it immediately.  This project has been evaluated and approved by the local ethics committee of the Department of Medicine at the University Hospital, LMU Munich and the local data protection officer. The data collection process is anonymous. The software has also been evaluated and approved by the local data protection officer of the University Hospital, LMU Munich. | | |
| **Please answer the following questions according to the provided scales.**  **This survey comprises 33 questions.** | | |
| **Question 1:** | **How old are you?** | |

| - 18 – 30 years | - 31 – 40 years | - 41 – 50 years |
| --- | --- | --- |
| - 51 – 60 years | - > 60 years |  |

| **Question 2** | **What is your gender?** |
| --- | --- |

| - Male | - Female | - divers (Mx) |
| --- | --- | --- |

| **Question 3:** | **What is your profession or field of profession?** |
| --- | --- |

| - Chief Physician | - Specialist | - Resident Physician |
| --- | --- | --- |
| - Nurse | - Researcher/Scientist | - Psychologist |
| - Social worker | - Non-medical therapeutic area | - Administration |

| Question 4: | What is your specialization in medicine? | | |
| --- | --- | --- | --- |
| - Internal Medicine | | - Anesthesiology | - Surgery |
| - Psychosomatic Medicine and Psychotherapy | | - Child and Youth Psychiatry and -Psychotherapy | - Psychiatry and Psychotherapy |
| - Obstetrics and Gynecology | | - Neurology | - Pediatrics |
| - Ophthalmology | | - ENT | - Urology |
| - Dermatology | | - Radiotherapy | - Nuclear Medicine |
| - Microbiology | | - Laboratory Medicine | - Radiology |
| - Other | |  |  |

| **Question 5:** | **Please specify on the extent of your personal professional medical experience (in years):** |
| --- | --- |

| - ≤ 3 years | - 4 – 6 years | - 7-10 years |
| --- | --- | --- |
| > 10 years  ***(this question was not analysed (see methods section)*** | | |

| **Question 6:** | **In which federal state of Germany** **do you work?** |
| --- | --- |

| - Bavaria | - Berlin | - Brandenburg |
| --- | --- | --- |
| - Baden-Württemberg | - Bremen | - Hesse |
| - Hamburg | - Lower Saxony | - Mecklenburg-Western Pomerania |
| - North Rhine-Westphalia | - Rhineland-Palatinate | - Saarland |
| - Saxony | - Saxony-Anhalt | - Schleswig-Holstein |
| - Thuringia |  |  |

| **Question 7:** | **Please specify the type of hospital setting in which you currently work.** |
| --- | --- |

| - University Hospital | - Public agency Hospital | - Non-profit carrier Hospital |
| --- | --- | --- |
| - Private carrier Hospital | - Other |  |

| **Question 8:** | **Please specify the type of hospital setting in which you currently work.** |
| --- | --- |

| - Ambulatory setting | - Emergency Room | - Normal ward |
| --- | --- | --- |
| - COVID-19-ward | - Intensive Care Unit | - not involved in patient care at all |

| **Question 9:** | **The COVID-19 pandemic has led to an increase in my daily workload.** | | | | | | |
| --- | --- | --- | --- | --- | --- | --- | --- |
| - strongly disagree | | - disagree | - neutral | | | - agree | - strongly agree |
| **Question 10:** | **Due to the COVID-19 pandemic I feel mentally strained.** | | | | | | |
| - strongly disagree | | - disagree | - neutral | | | - agree | - strongly agree |
| **Question 11:** | **My superiors/my employer informed me sufficiently about COVID-19.** | | | | | | |
| - strongly disagree | | - disagree | - neutral | | | - agree | - strongly agree |
| **Question 12:** | **Since the outbreak of the COVID-19 pandemic, the satisfaction with my job has worsened.** | | | | | | |
| - strongly disagree | | - disagree | - neutral | | | - agree | - strongly agree |
| **Question 13:** | **I feel left alone by my employer.** | | | | | | |
| - strongly disagree | | - disagree | - neutral | | | - agree | - strongly agree |
| **Question 14:** | **I feel left alone by the responsible political decision-makers.** | | | | | | |
| - strongly disagree | | - disagree | - neutral | | | - agree | - strongly agree |
| **Question 15:** | **I belong to the Covid-19 high-risk group (due to pre-existing conditions, age)** | | | | | | |
| - Yes | | - No |  | | |  |  |
| **Question 16:** | **How many patients tested positive for COVID-19 have you treated until now?** | | | | | | |
| Please provide a number: | | | |  |  | | |
| **Question 17:** | **How many close friends or family members have already been tested positive for COVID-19?** | | | | | | |
| Please provide a number: | | | |  |  | | |
| **Question 18:** | **Have you been tested positive for COVID-19?** | | | | | | |
| - Yes | | - No |  | | |  |  |
| **Question 19:** | **The measures taken by the hospital administration have been appropriate (in terms of supply with information, protective equipment, organization of work processes).** | | | | | | |
| - strongly disagree | | - disagree | - neutral | | | - agree | - strongly agree |
| **Question 20:** | **In my opinion, the communication related to COVID-19 that came from the management of the hospital has been appropriate.** | | | | | | |
| - strongly disagree | | - disagree | - neutral | | | - agree | - strongly agree |
| **Question 21:** | **I have the impression that my efforts at work during the Covid-19 pandemic are being appreciated by the management of the hospital.** | | | | | | |
| - strongly disagree | | - disagree | - neutral | | | - agree | - strongly agree |
| **Question 22:** | **My hospital was/is well prepared with regard to the Covid-19 pandemic** | | | | | | |
| - strongly disagree | | - disagree | - neutral | | | - agree | - strongly agree |
| **Question 23:** | **On average, how many additional overtime hours per week are you working since the outbreak of the Covid-19 pandemic?** | | | | | | |
| Please provide a number: | | | |  | hours/week | | |
| **Question 24:** | **Due to the COVID-19 pandemic, I have significantly less time for my personal life.** | | | | | | |
| - strongly disagree | | - disagree | - neutral | | | - agree | - strongly agree |
| **Question 25:** | **My daily life has become more stressful due to the COVID-19 pandemic.** | | | | | | |
| - strongly disagree | | - disagree | - neutral | | | - agree | - strongly agree |
| **Question 26:** | **Due to the COVID-19 pandemic, I am worrying more often about the future.** | | | | | | |
| - strongly disagree | | - disagree | - neutral | | | - agree | - strongly agree |
| **Question 27:** | **Due to the COVID-19 pandemic I am worrying more often about the well-being of my family.** | | | | | | |
| - strongly disagree | | - disagree | - neutral | | | - agree | - strongly agree |
| **Question 28:** | **I am afraid of catching the Coronavirus myself.** | | | | | | |
| - strongly disagree | | - disagree | - neutral | | | - agree | - strongly agree |
| **Question 29:** | **I fear that due to my daily exposure with it at work, I could pass on the coronavirus to my friends or relatives.** | | | | | | |
| - strongly disagree | | - disagree | - neutral | | | - agree | - strongly agree |
| **Question 30:** | **Since the COVID-19 pandemic, I have been sleeping less well.** | | | | | | |
| - strongly disagree | | - disagree | - neutral | | | - agree | - strongly agree |
| **Question 31:** | **In my setting, patients not infected with Covid-19 are adequately taken care of despite the Covid-19 pandemic.** | | | | | | |
| - strongly disagree | | - disagree | - neutral | | | - agree | - strongly agree |
| **Question 32:** | **In my hospital setting, Covid-19 positive patients are adequately taken care of.** | | | | | | |
| - strongly disagree | | - disagree | - neutral | | | - agree | - strongly agree |
| **Question 33:** | **I will continue to work in the healthcare area after the Covid-19 pandemic.** | | | | | | |
| - strongly disagree | | - disagree | - neutral | | | - agree | - strongly agree |
